# Recurrent “transient ischemic attacks” caused by pulmonary arteriovenous malformation

**DOI:** 10.1016/j.rmcr.2025.102172

**Published:** 2025-01-25

**Authors:** Jorrit B.A. Welling, Mascha Schuurmans, T. David Koster

**Affiliations:** a University of Groningen, University Medical Center Groningen, Department of Pulmonary Diseases, Groningen, the Netherlands; b Groningen Research Institute for Asthma and COPD, University of Groningen, University Medical Center Groningen, Groningen, the Netherlands; c Department of Neurology, Medical Center Leeuwarden, Leeuwarden, the Netherlands

**Keywords:** Arteriovenous malformation, Transient ischemic attack

## Abstract

Pulmonary arteriovenous malformations (PAVMs) are abnormal, direct connections between a pulmonary artery and pulmonary vein that can lead to severe neurologic complications including ischemic stroke. This case report describes a 39-year-old woman who presented with recurrent transient ischemic attacks (TIAs). Extensive clinical evaluation revealed PAVM as the underlying cause. Following successful embolization of the PAVM, the patient did not experience further TIAs. This case highlights the importance of considering PAVMs in the differential diagnosis of recurrent TIAs, particularly in patients without traditional risk factors for cerebrovascular events.

## Highlights

• A PAVM is an abnormal, direct connection between a pulmonary artery and pulmonary vein.
• PAVMs are a rare cause for transient ischemic attacks but should not be overlooked, especially in younger patients without traditional cerebrovascular risk factors.
• Embolization of PAVM carries minimal risks and is an effective way to reduce risk of cerebrovascular events.

## 1 Introduction

Transient ischemic attacks (TIAs) are brief episodes of neurological dysfunction caused by focal brain ischemia without evidence of acute infarction on brain imaging [1]. Common causes of TIAs include large artery atherosclerosis, cardio embolism and small-vessel occlusion [2].

A pulmonary arteriovenous malformation (PAVM) is an abnormal, direct connection between a pulmonary artery and pulmonary vein, resulting in a right to left shunt. Since blood that flows through the PAVM bypasses the pulmonary capillary network, there is no filter function for thrombotic and septic emboli, increasing the risk of TIA, ischemic stroke and brain abscess [3]. PAVMs can also lead to hypoxemia due to right-to-left shunting and when ruptured may cause hemoptysis or hemothorax [4].

## 2 Patient presentation

A 39-year-old woman, without prior medical history, presented multiple times to the emergency department with transient symptoms of aphasia, dysarthria and a tingling sensation in her right arm. The patient had no significant medical history, was a never-smoker, and had no known cardiovascular risk factors. Apart from the symptoms related to the TIA's, she did not experience any other symptoms, especially no epistaxis, hemoptysis or exertional dyspnea. No platypnea-orthodeoxia, clubbing, or mucocutaneous telangiectasia were observed during the physical examination. Arterial blood gas analysis was performed on room air which demonstrated a pO2 of 10.6 kPa (in rest), neither anemia or polycythemia was observed. An electrocardiogram did not reveal any arrhythmias. A brain MRI scan was performed which demonstrated laesions compatible with old ischemia. The patient was diagnosed with recurrent transient ischemic attacks (TIAs).

## 3 Investigations

**Fig. 1:**
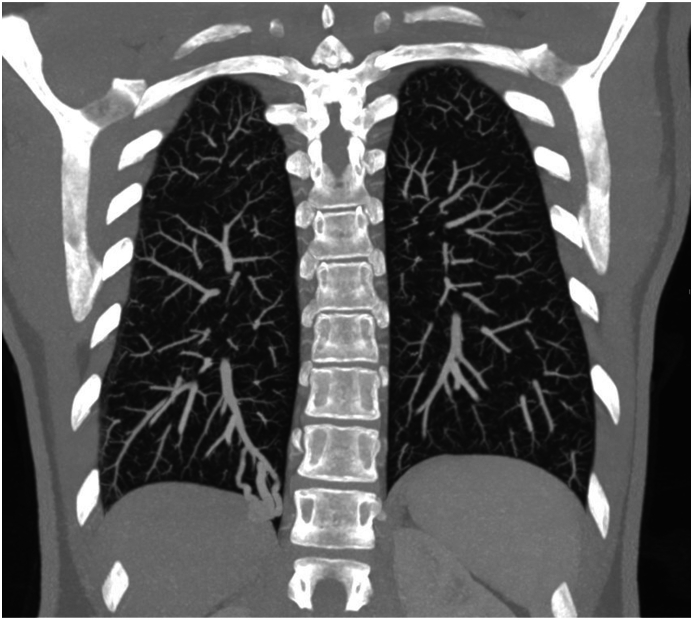
Coronal Maximal Intensity Projection (MIP) Computed Tomography image of the chest of a 39-year-old female with recurrent transient ischemic attacks, a pulmonary arteriovenous malformation is present in the right lower lobe.

Given the recurrent nature of the TIAs and the absence of traditional risk factors, a more thorough investigation was initiated. The patient underwent a bubble contrast transesophageal echocardiogram (TEE), more than 30 bubbles appeared in the left atrium after 6 cardiac cycles, demonstrating right-to-left shunting and suggesting a possible intracardiac or pulmonary source of embolism. Initially, a patent foramen ovale (PFO) was thought to be the cause of right-to-left shunting and the patient was scheduled for PFO closure. However, during the PFO closure procedure, no PFO could be identified and a cardiac cause of right to left shunt was ruled out. A subsequent CT scan of the thorax with intravenous contrast was performed which demonstrated a PAVM in the right lower lobe, with a feeding artery diameter of 6mm (Fig. 1).

The patient did not meet the diagnostic criteria for hereditary hemorrhagic telangiectasia (HHT), also known as Osler-Weber-Rendu syndrome, a condition that is frequently associated with PAVM. For the diagnosis of HHT, the Curacao criteria can be used, and this patient only met one out of four criteria (visceral AVM) and did not experience any nose bleeds, had no telangiectatic lesions and no family history of HHT. When fewer than 2 of the Curacao criteria are met, the diagnosis HHT is considered unlikely [5]. The patient was referred for genetic testing and no genetic mutations in *ENG, ACVRL1, SMAD4* en *GDF2* genes were found [6].

## 4 Management

**Fig. 2:**
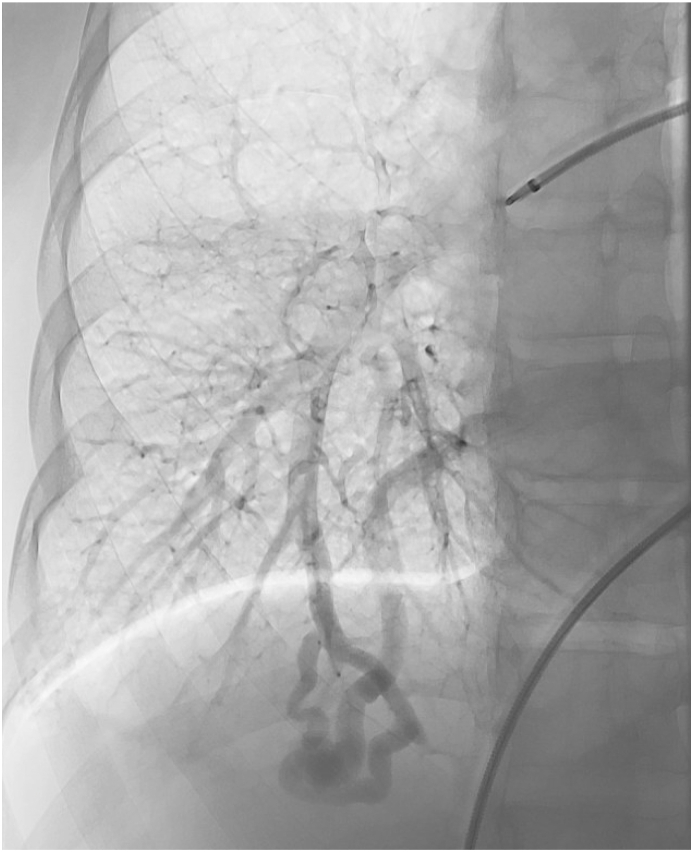
Pulmonalis angiography of a 39-year-old female with recurrent transient ischemic attacks, a pulmonary arteriovenous malformation is present in the right lower lobe.

The decision was made to proceed with percutaneous embolization of the PAVM. The procedure was performed under general anesthesia, with selective closure of the feeding artery supplying the AVM with an Amplatzer vascular plug (Fig. 2). The patient tolerated the procedure well and had an uneventful recovery. Notably, the patient did not experience any further recurrence of neurological symptoms after the embolization.

## 5 Discussion

This case highlights the uncommon but important association between pulmonary arteriovenous malformation and TIAs. TIAs secondary to pulmonary arteriovenous malformations (PAVM) are rare but should not be overlooked, especially in younger patients without conventional risk factors for cerebrovascular disease. PAVM related stroke, when compared to ischemic stroke of other causes, is associated with younger age at presentation, more disability adjusted life years and more years of life lost [7]. Radiological evidence of cerebral infarction was observed in up to 50 % of asymptomatic patients with PAVM [8]. In patients with an increased prevalence of PAVM, such as HHT, contrast enhanced transthoracic echocardiography can be used for screening and a chest CT scan with or without contrast can be performed to confirm the presence of PAVM and identify its characteristics [9,10]. In asymptomatic patients with a PAVM feeding artery diameter <2mm, follow-up can be considered instead of directly proceeding to embolization [10,11]. Limited evidence suggests that approximately 25 % of PAVM grow slowly over years [12,13].

The primary treatment for symptomatic PAVMs and PAVMs at risk for complications, is percutaneous transcatheter embolization. Embolization is a minimally invasive procedure that involves occluding the feeding vessels of the AVM using coils, plugs, or other devices [14]. Embolization of PAVM carries minimal risks and is an effective way to reduce risk of cerebrovascular events, cerebral abscesses, improve oxygenation in case of right to left shunting and treat PAVM induced hemoptysis [4]. A potential contra-indication for embolization of PAVM is severe pulmonary hypertension, as this group is at risk for increasing pulmonary arterial pressure after embolization and potential subsequent right heart failure [15,16]. Follow-up with either CT or transthoracic contrast echocardiography is suggested after embolization, as recanalization of treated PAVMs can occur, and should be performed within 6–12 months of treatment, then repeated every 3 years [17,18]. Patients with PAVM should be recommended antibiotic prophylaxis for procedures with risk of bacteremia and when intravenous access is obtained, extra care should be taken to avoid intravenous air [19].

## 6 Conclusion

This case underscores the importance of considering PAVMs in the differential diagnosis of recurrent TIAs, especially in younger patients without traditional cerebrovascular risk factors.

## CRediT authorship contribution statement

**Jorrit B.A. Welling:** Writing – review & editing, Writing – original draft, Conceptualization. **Mascha Schuurmans:** Writing – review & editing, Conceptualization. **T. David Koster:** Writing – review & editing, Writing – original draft, Conceptualization.

## Consent

Written informed consent was obtained from the patient for publication of the details of their medical case and any accompanying images.

## Ethical approval

Ethical approval was not required for this case report, however written informed consent was obtained from the patient and is available for review under request.

## Data availability statement

Not applicable.

## Funding sources

Not applicable.

## Declaration of competing interest

The authors declare that they have no known competing financial interests or personal relationships that could have appeared to influence the work reported in this paper.
